# Arterial blood pressure monitoring in overweight critically ill patients: invasive or noninvasive?

**DOI:** 10.1186/cc4896

**Published:** 2006-04-19

**Authors:** Ali Araghi, Joseph J Bander, Jorge A Guzman

**Affiliations:** 1 Division of Pulmonary, Critical Care, and Sleep Medicine, Wayne State University School of Medicine, Detroit, Michigan, USA

## Abstract

**Introduction:**

Blood pressure measurements frequently guide management in critical care. Direct readings, commonly from a major artery, are considered to be the gold standard. Because arterial cannulation is associated with risks, alternative noninvasive blood pressure (NIBP) measurements are routinely used. However, the accuracy of NIBP determinations in overweight patients in the outpatient setting is variable, and little is known about critically ill patients. This prospective, observational study was performed to compare direct intra-arterial blood pressure (IABP) with NIBP measurements obtained using auscultatory and oscillometric methods in overweight patients admitted to our medical intensive care unit.

**Method:**

Adult critically ill patients with a body mass index (BMI) of 25 kg/m^2 ^or greater and a functional arterial line (assessed using the rapid flush test) were enrolled in the study. IABP measurements were compared with those obtained noninvasively. A calibrated aneroid manometer (auscultatory technique) with arm cuffs compatible with arm sizes and a NIBP monitor (oscillometric technique) were used for NIBP measurements. Agreement between methods was assessed using Bland-Altman analysis.

**Results:**

Fifty-four patients (23 males) with a mean (± standard error) age of 57 ± 3 years were studied. The mean BMI was 34.0 ± 1.4 kg/m^2^. Mean arm circumference was 32 ± 0.6 cm. IABP readings were obtained from the radial artery in all patients. Only eight patients were receiving vasoactive medications. Mean overall biases for the auscultatory and oscillometric techniques were 4.1 ± 1.9 and -8.0 ± 1.7 mmHg, respectively (*P *< 0.0001), with wide limits of agreement. The overestimation of blood pressure using the auscultatory technique was more important in patients with a BMI of 30 kg/m^2 ^or greater. In hypertensive patients both NIBP methods underestimated blood pressure as determined using direct IABP measurement.

**Conclusion:**

Oscillometric blood pressure measurements underestimated IABP readings regardless of patient BMI. Auscultatory measurements were also inaccurate, tending to underestimate systolic blood pressure and overestimate mean arterial and diastolic blood pressure. NIBP can be inaccurate among overweight critically ill patients and lead to erroneous interpretations of blood pressure.

## Introduction

Although the prevalence of critically ill, morbidly obese patients in the USA is not known, it has been estimated that the incidence of morbidly obese patients requiring intensive care treatment approaches 14 cases per 1,000 intensive care unit (ICU) admissions each year. This is probably a conservative estimate, considering that the database was restricted to nonsurgical patients and the growing number of bariatric surgeries performed in the USA [1]. Obese patients in the ICU face a more complicated course, and their obesity has impacts on various aspects of their care. Obesity makes hemodynamic monitoring more challenging because of difficulties with inserting intravascular catheters and unsuited or inappropriate cuff-to-arm sizes [1,2]. Discrepancies between direct intra-arterial blood pressure (IABP) and indirect noninvasive blood pressure (NIBP) measurements can adversely affect therapeutic decisions and may have a negative impact on outcomes.

Because of the frequent need for prolonged monitoring of blood pressure among critically ill patients, automated oscillometric NIBP measurements are commonly used in the ICU [3,4]. Sources of error and accuracy problems associated with age, presence of arrhythmias, inaccurate cuff selection and positioning, and rapid cuff deflation have been described [5,6]. Among obese patients the auscultatory technique for NIBP measurement underestimates systolic blood pressure and overestimates diastolic blood pressure, but very few data exist regarding the accuracy of automated oscillometric measurements [7,8].

The present observational clinical study was conducted to test the hypothesis that IABP measurements are not accurately reflected by NIBP measurements in a population of overweight critically ill patients. We also assessed the effects of different body mass index (BMI) and blood pressure levels on the accuracy of the NIBP measurements when compared with direct IABP readings.

## Materials and methods

The study protocol was approved by the Wayne State University investigational review board. All patients admitted to the medical ICU with a BMI of 25 kg/m^2 ^or greater who required continuous blood pressure monitoring because of their underlying clinical condition and who agreed to participate in the study were included. None of the patients received an intra-arterial catheter purely for the purposes of the present study. Patients with any of the following criteria/conditions were excluded: BMI below 25 kg/m^2^; unwillingness to participate in the study; unstable IABP readings (more than 5 mmHg variation in mean blood pressure during the period of data collection); presence of edema, wounds, or arm skin or subcutaneous tissue infection; presence of a peripherally inserted central catheter in one arm; and nonfunctional arterial catheter (defined as presence of overshooting or undershooting phenomenon following rapid flush test) [9].

Each patient's height, weight, and arm circumferences at the mid-arm level were recorded. After being in a steady, supine position, auscultatory and oscillometric blood pressure measurements were obtained from the arm into which the arterial catheter was inserted [10]. The corresponding intra-arterial reading was obtained immediately at the end of each noninvasive measurement and averaged for the purposes of data analysis.

### Invasive blood pressure measurements

Arterial catheterization was performed by the primary team according to their determination of the clinical indication. A 20-gauge radial artery set (Arrow International, Reading, PA, USA) was used for continuous IABP monitoring. The sets were connected to a disposable pressure transducer (TruWave, Edwards Lifesciences, Irvine, CA, USA) using rigid pressure tubing of identical length. The transducer set system was set up by the critical care nurse and checked by the investigators in all cases. Air bubbles were flushed carefully from the system before data collection. To test the adequacy of the pressure monitoring system, a rapid flush test was performed and recorded for each patient [9]. The zero level for arterial blood pressure was taken at the right atrium level and the arterial wave form was recorded from the monitor (Hewlett-Packard, model 66, Andover, MA, USA).

### Noninvasive blood pressure monitoring

Oscillometric measurements were obtained using a Hewlett-Packard monitor (model 66) and nondisposable blood pressure cuffs (Philips Medical Systems, Andover, MA, USA) models M4554A, M1575A, and M1576A to match arm circumferences ranging from 20.5 to 28.5 cm, from 34 to 43 cm, and from 42 to 54 cm, respectively. Auscultatory measurements were obtained using an aneroid manometer (Econosphyg, McCoy, USA) with reusable blood pressure cuffs (Critikon Dura-Cuf models 2203, 2204, and 2205; GE Medical System, Waukesha, WI, USA) matching the patients' arm circumferences. Standard recommendations for cuff bladder length-to-width ratio and technical details and cautions before and during measurements were strictly followed [11,12]. All auscultatory measurements were obtained by the same investigator (AA).

### Statistical methods

Unless stated otherwise, summary values are expressed as mean ± standard error. Different methods of blood pressure measurement were compared by Bland-Altman analysis [13]. Unpaired Student's t test was used to compare mean differences between radial and femoral sites; blood pressure readings in patients with BMI >25 kg/m^2^, ≤30 kg/m^2^, and >30 kg/m^2^; and systolic blood pressure below or above 140 mmHg for each NIBP method. A two tailed *P *value of less than 0.05 was considered statistically significant.

## Results

Fifty-four patients (23 males and 24 females) with mean age 57 ± 3 years who were admitted to the medical ICU requiring invasive blood pressure monitoring were included in the study. Mean BMI was 34.0 ± 1.4 kg/m^2 ^(range 25–87.2 kg/m^2^) and mean arm circumference was 32.4 ± 0.6 cm (right and left arms were similar). An adult size cuff (suitable for arm circumferences <42 cm) was used in all but seven patients, in whom a large cuff was used based on their arm circumferences (41.6 ± 1.5 cm). All direct IABP measurements were obtained from radial lines, and only eight patients were receiving vasoactive medications during data collection.

**Figure 1:**
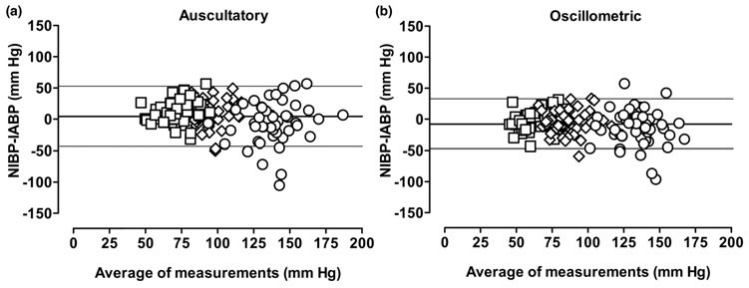
Agreement between auscultatory and oscillometric, and intra-arterial blood pressure measurements. Shown are graphs for the **(a) **auscultatory method and **(b) **oscillometric method. The thicker line represents mean bias, and the thinner lines represent upper and lower limits of agreement. Squares are diastolic blood pressure measurement, diamonds are mean arterial blood pressure measurements, and circles are systolic blood pressure measurements. *P *< 0.001 between methods. IABP, intra-arterial blood pressure; NIBP, noninvasive blood pressure. a and b are not identified in the figure.

Plots of agreement between the auscultatory and oscillometric methods and IABP measurements (54 patients, 162 pairs of measurements in each plot) are shown in Figure 1. There was a statistically significant discrepancy between the auscultatory and oscillometric arterial blood pressure measurement (mean biases 4.1 ± 1.9 and -8.0 ± 1.7 mm Hg, respectively; *P *< 0.0001), with limits of agreement ranging from +53.0 to -44.6 mmHg and from +33.6 to -49.5 mmHg for auscultatory and oscillometric methods, respectively. Most of the points below the lower limit of agreement seen in both plots represent patients who were receiving vasopressor infusions. Discrepancies according to arterial blood pressure levels are shown in Table 1. Overall, oscillometric measurements underestimated IABP (mainly systolic blood pressure) measurements. On the other hand, the auscultatory method underestimated systolic and overestimated diastolic and mean arterial blood pressure measurements.

**Figure 2:**
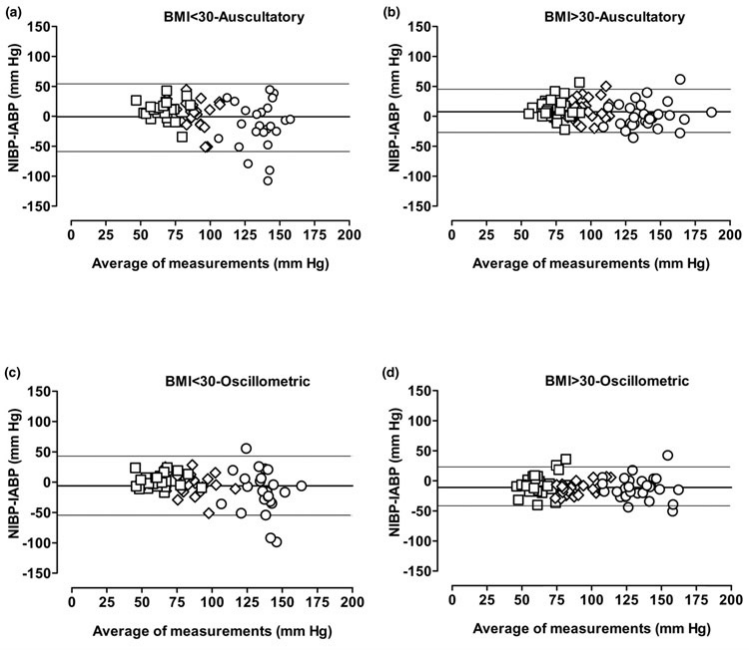
Agreement between auscultatory and oscillometric, and intra-arterial blood pressure measurements: effect of BMI. Shown are graphs of measurement in **(a) **overweight (BMI ≤30 kg/m^2^) **(b) **obese (BMI >30 kg/m^2^) patients by auscultatory technique, and **(c) **overweight and **(d) **obese patients by oscillometric technique. The thicker line represents mean bias, and the thinner lines represent upper and lower limits of agreement. Squares are diastolic blood pressure measurement, diamonds are mean arterial blood pressure measurements, and circles are systolic blood pressure measurements. BMI, body mass index; IABP, intra-arterial blood pressure; NIBP, noninvasive blood pressure.

Figure 2 shows plots of agreement between the NIBP (auscultatory and oscillometric) methods and IABP measurements after the cohort was divided according to BMI. Mean biases for the auscultatory method were -0.8 ± 3.6 and 7.6 ± 2.1 mmHg for patients with a BMI ≤30 kg/m^2 ^and >30 kg/m^2^, respectively (*P *< 0.05), whereas measurements were similar for the oscillatory method regardless of BMI. Table 2 shows biases for each monitoring method according to BMI and for each level of blood pressure separately. Large discrepancies between patient groups were observed for systolic and mean arterial blood pressure when the auscultatory method was evaluated and for mean and diastolic pressures when the oscillometric method was studied.

**Figure 3:**
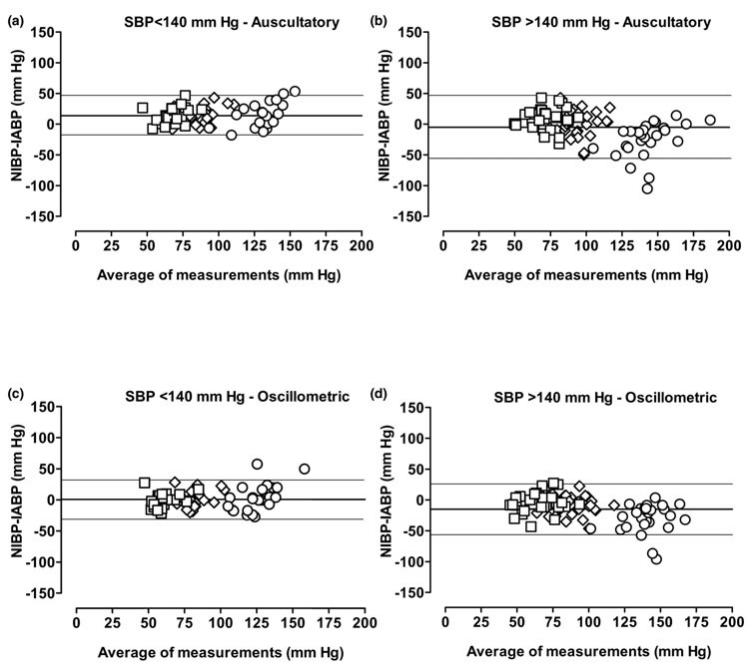
Agreement between auscultatory and oscillometric, and intra-arterial blood pressure measurements: effect of SBP. Shown are graphs of measurement in patients with **(a) **SBP <140 mmHg and **(b) **SBP ≥140 mmHg by auscultatory technique, and in patients with **(c) **SBP <140 mmHg and **(d) **SBP ≥140 mmHg by oscillometric technique. A total of 23 patients had SBP <140 mmHg and 31 had SBP ≥140 mmHg. The thicker line represents mean bias, and the thinner lines represent upper and lower limits of agreement. Squares are diastolic blood pressure measurement, diamonds are mean arterial blood pressure measurements, and circles are systolic blood pressure measurements. IABP, intra-arterial blood pressure; NIBP, noninvasive blood pressure; SBP, systolic blood pressure.

The effects of blood pressure levels on accuracy of NIBP measurements are shown in Figure 3. A cut off of 140 mmHg systolic blood pressure level was selected to divide the cohort into two groups. Both methods underestimated IABP measurements among patients with a systolic blood pressure above 140 mmHg (mean bias of -5.0 ± 2.8 mmHg and -14.9 ± 2.2 mmHg for the auscultatory and oscillometric methods, respectively; *P *< 0.001). On the other hand, mean bias was positive for both NIBP methods in patients with normal blood pressure.

## Discussion

Accurate measurement of arterial blood pressure is essential for rational hemodynamic management of critically ill patients. Morbidly obese patients requiring intensive care treatment are becoming increasingly common and, because of their body size and habitus, it is unclear whether invasive and noninvasive blood pressure measurements could be used interchangeably. Our data suggest that a wide discrepancy exists between blood pressure monitoring methods, supporting the use of direct intra-arterial methods in monitoring and to guide treatment decisions because of their accuracy.

### Overall bias

Oscillometric measurements underestimated direct intra-arterial readings, and although this was observed for all levels of blood pressure the negative bias was larger for systolic blood pressure measurements. Although our study focused on overweight critically ill patients, these findings are in agreement with those of previously reported trials conducted in different patient populations [6,14,15]. Because the oscillometric method is not standardized, measuring algorithms differ from manufacturer to manufacturer and even from device to device [10,16]. The variability in these empirically derived algorithms has been blamed for the lack of agreement between methods, and although the use of a new standardized algorithm decreased the negative bias, oscillometric measurements continued to underestimate IABP readings [14]. Inappropriateness of cuff sizes in relation to arm circumference is also responsible for underestimation or overestimation of direct blood pressure readings [4,10]. It is unlikely that this was a factor in our study because the most common mismatch for obese patients is a smaller than needed blood pressure cuff, yielding an overestimated NIBP reading. Moreover, the intentional use of a smaller than recommended cuff size has been postulated to decrease the negative biases attributed to oscillometric readings [14]. Arrhythmias, another factor that is known to cause inaccurate oscillometric blood pressure readings, were not present at the time of data collection. Furthermore, inotropic support did not contribute to the inaccuracy of the measurements in a larger group of patients [14], and although the points below the lower limits of agreement corresponded to patients receiving vasopressor support, these few observations are not likely to be responsible for the overall negative bias observed in our study.

The mean bias for the auscultatory technique was 4.2 mmHg, but when broken down for each level of pressure our data are consistent with previous observations, namely that auscultatory measurements underestimate systolic and overestimated diastolic blood pressure readings [5,7,8]. The upward bias in diastolic blood pressure produced by cuff inflation probably relates to increased blood volume in the arm distal to the cuff while cuff pressure still exceeds venous pressure and occludes venous return. This would impair diastolic run-off of blood and elevate diastolic pressure [17].

### Body mass index

BMI above 30 kg/m^2 ^had little impact on the overall findings. Oscillometric measurements consistently underestimated direct blood pressure measurements. On the other hand, our findings are in agreement with those of previous investigations demonstrating that auscultatory readings overestimated diastolic blood pressure [7,8]. Mean bias for systolic pressure readings by the auscultatory method became slightly positive in patients with BMI above 30 kg/m^2^. Although hypothetical, an inability to properly position the blood pressure cuff in these large patients might have resulted in a bad signal/noise ratio, which could account for these findings [10].

### Effects of hypertension

Arterial hypertension increased the negative bias for systolic blood pressure measurements for both noninvasive monitoring methods. This observation has been reported for the auscultatory technique [7,8] and appears to be explained not by an inability to record the first audible Korotkoff sound but by the increasing critical closing pressure with increasing levels of blood pressure [8].

## Conclusion

Although widely used, automated oscillometric measurements of blood pressure were inaccurate in this subset of critically ill patients, and the parameters obtained should be used cautiously. When critical therapeutic decisions are required, IABP monitoring may be the preferred monitoring method.

## Key messages

• NIBP measurements are inaccurate among overweight critically ill patients.

• Oscillometric NIBP measures underestimates blood pressure as determined using the direct IABP technique.

• When critical therapeutic decisions are required, IABP monitoring is the preferred monitoring method.

## Abbreviations

BMI = body mass index; IABP = intra-arterial blood pressure; ICU = intensive care unit; NIBP = noninvasive blood pressure.

## Competing interests

The authors declare that they have no competing interests.

## Authors' contributions

AA was involved in design, data collection, and manuscript preparation. JB was involved in manuscript drafting and critical revisions. JG was involved in data analysis and interpretation, manuscript drafting, and critical manuscript revisions. All authors read and approved the final manuscript.

## Figures and Tables

**Table 1:** Biases and limits of agreement between blood pressure measurement techniques for each level of blood pressure

Arterial blood pressure	Mean bias	Upper limit of agreement	Lower limit of agreement
Systolic blood pressure (mmHg)			
Auscultatory	-6.7 ± 4.3	55.1	-68.5
Oscillometric	-15.2 ± 3.7	37.7	-68.2
Diastolic blood pressure (mmHg)			
Auscultatory	11.4 ± 2.3	44.8	-22.1
Oscillometric	-3.7 ± 2.0	25.5	-33.0
Mean blood pressure (mmHg)			
Auscultatory	7.9 ± 2.7	47.6	-31.6
Oscillometric	-4.6 ± 2.5	31.1	-40.3

Values are expressed as mean ± standard error.

**Table 2:** Biases and limits of agreement between blood pressure measurement techniques for each level of blood pressure: effect of BMI. Parenthesis indicate upper and lower limits of agreement

Arterial blood pressure	BMI <30 kg/m^2 ^(*n *= 24)	BMI >30 kg/m^2 ^(*n *= 30)
Systolic (mmHg)		
Auscultatory	-16.0 ± 8.1 (+61.8 to -93.8)	0.1 ± 3.8 (+40.8 to -40.7)
Oscillometric	-16.7 ± 7.3 (+53.0 to -86.4)	-13.3 ± 3.4 (+23.1 to -49.6)
Diastolic (mmHg)		
Auscultatory	10.0 ± 3.3 (+41.6 to -21.5)	11.9 ± 2.9 (+43.7 to -20.0)
Oscillometric	1.3 ± 2.3 (+23.4 to -20.9)	-7.8 ± 2.9 (+24.2 to -39.8)
Mean (mmHg)		
Auscultatory	3.7 ± 4.7 (+48.7 to -44.4)	11.2 ± 3.0 (+43.7 to -21.2)
Oscillometric	-2.3 ± 3.2 (+32.5 to -37.5)	-12.3 ± 1.7 (+6.7 to -31.3)

Values are expressed as mean ± standard error.
